# Probability of a timely vocal response in mother-infant interaction and later psychiatric diagnosis: A case-control study

**DOI:** 10.1371/journal.pone.0344552

**Published:** 2026-07-01

**Authors:** Bethany Stanley, Clare S. Allely, Jenna Charlton, Christopher Gillberg, James Law, Penny Levickis, Alex McConnachie, Christine Puckering, Lucy Thompson, Philip Wilson

**Affiliations:** 1 Robertson Centre for Biostatistics, School of Health and Wellbeing, University of Glasgow, Glasgow, United Kingdom; 2 School of Health and Society, Centre for Applied Health Research, University of Salford, Salford, United Kingdom; 3 School of Education, Communication and Language Sciences, Newcastle University, Newcastle upon Tyne, United Kingdom; 4 Gillberg Neuropsychiatry Centre, Institute of Neuroscience and Physiology, Sahlgrenska Academy, University of Gothenburg, Gothenburg, Sweden; 5 Department of Child and Adolescent Psychiatry, School of Health and Wellbeing, Royal Hospital for Sick Children, Glasgow, United Kingdom; 6 REEaCh Centre, Faculty of Education, The University of Melbourne, Melbourne, Victoria, Australia; 7 Institute of Applied Health Sciences, University of Aberdeen, Inverness, United Kingdom; 8 Department of General Practice, University of Copenhagen, Copenhagen, Capital Region, Denmark; Hacettepe University: Hacettepe Universitesi, TÜRKIYE

## Abstract

Patterns of parent-child interactions are commonly cited as being predictive of later psychiatric disorders but precisely which elements of these interactions are important is rarely clear, potentially affecting the effective targeting of interventions in young children. The current study aimed to examine the relationship between timely vocal response during parent-child interactions (i.e., the probability of mothers responding to their child within a specified time period and vice versa), and later psychiatric diagnosis. Drawing on data from the Avon Longitudinal Study of Parents and Children (ALSPAC) cohort, a case control study was conducted based on infant-mother video observations of children assessed for neuropsychiatric disorders using the parent-reported Development and Wellbeing Assessment (DAWBA) at seven years of age (103 controls and 55 cases). Empirical examination suggested that 1 second represented the optimal threshold for maternal responses and 8 seconds for child responses. Only the maternal measure was found to predict later psychiatric disorders, with evidence of associations limited to hyperactivity and conduct disorders. These associations were not sensitive to either maternal education or child sex. The results are discussed in terms of the value of precise interpretation of early mother/child interaction and for the potential for providing targeted intervention to the population concerned.

## Introduction

Psychiatric disorders of childhood and adolescence are among the major causes of disability worldwide and are risk factors for premature mortality [1,2]. A substantial body of evidence demonstrates that early social interactions between parents and children predict important aspects of child social, emotional and behavioural development [1–3] as well as specific psychiatric diagnoses [4–7]. A key mechanism underlying these associations is temporal contingency in early parent-child interactions, defined as the timing with which parents and infants respond to one another’s vocalisations and behaviours. Temporally contingent parental responses provide infants with predictable feedback that supports the development of early emotion regulation, social competence, and communication skills that lay the foundation for later mental health [11,12]. In contrast, low levels of maternal responsiveness [8–11], reduced sensitivity to infant cues [6] and limited involvement in play [12] have been consistently associated with child behavioural and social-emotional difficulties. Infant characteristics may also contribute to poorer outcomes, with early emotional dysregulation [13], social withdrawal [14] and quality of vocalisations [15–18] predicting later psychopathology. This underscores the inherently bidirectional nature of parent-child interactions, whereby child behaviours can influence parental responses just as parenting behaviours can influence child social, emotional and behavioural development [19]. Within this bidirectional framework, response timing represents a measurable indicator of how effectively parents and infants coordinate their behaviour, which could be used in identifying children at risk of later psychiatric disorder.

The significance of early parent-child interactions for later development can be further understood through attachment theory, which posits that sensitive and responsive caregiving fosters secure attachment, providing infants with a reliable foundation from which to explore the world and regulate their emotions [20,21]. Research has shown that high maternal sensitivity which promotes secure attachment is characterised by mothers responding promptly to their infant’s behaviours [22,23]. Securely attached children are more likely than their peers to develop social competence and resilience, whereas inconsistent or delayed caregiver responses may contribute to unsecure attachment, increasing the risk of behavioural and emotional difficulties. Complementing this is social learning theory which emphasises the role of observational learning and reinforcement early in development [24]. Infants learn about social contingencies, emotional regulation and appropriate behaviours through repeated and consistent interactions with caregivers, with temporally contingent responses providing immediate reinforcement that contributes to future behaviour [25].

A number of intrinsic and extrinsic risk factors contribute to the nature of parent-child interactions, including biological, social, psychological and socio-demographic factors. Frequently documented are the impacts of psychosocial factors such as maternal mental health [26,27] and socio-demographic factors such as maternal age and education [28,29]. Socio-economic status (SES) is also associated with interaction quality and quantity, with parents from higher SES backgrounds demonstrating greater responsiveness and sensitivity towards their children, less controlling interaction styles, greater cognitive stimulation and talking more with children [30,31]. Documented across these factors, language and communication emerge as key mediators of interaction quality, highlighting the importance of examining not only how much parents and infants vocalise, but how these vocalisations are temporally coordinated.

Therefore, response timing is a particularly informative component of maternal sensitivity to examine, as it captures the predictability of parent-infant coordination beyond global measures of responsiveness [26]. Timely parental responses can reinforce positive behaviours and support infant’s developing emotional regulation, fostering secure attachment and social development [23]. Conversely, delayed responses can contribute to misunderstandings and emotional distress, potentially leading to behavioural difficulties and psychiatric disorders.

Identifying early predictors of later childhood psychiatric disorder in the vocalisations between caregivers and infants may assist in the early detection of children at risk of developing disorders, and potentially could allow earlier intervention. Speech and vocalisation metrics are potentially valuable objective indicators for a range of early psychiatric disorders but relatively few studies have collected prospective data capturing both early social interaction and later diagnoses in a population-based sample. One such study is the Avon Longitudinal Study of Parents and Children (ALSPAC) cohort [5,6,27]. Previous work based on this cohort has indicated that low levels of parental activity [5], speech [5,27] and responsiveness to infant cues [6] predicted psychiatric disorder, regardless of the presence of maternal depression.

The present study builds on the previous examination of vocalisations in early caregiver-infant interactions within the ALSPAC cohort and later childhood psychiatric disorders [5,27]. In contrast to simple metrics based on the average number and duration of vocalisations, we attempted to capture maternal and child vocal response by evaluating the probability of the mother responding to the child within an appropriate time period and the corresponding probability of the child responding. This study therefore aimed to address the following questions:

1) To what extent is the vocal response (i.e., the probability of the mother or child responding in a specified time period to the other) a key predictive component of mother-infant interaction and where should the response time thresholds lie?
2) To what extent does vocal response (mother and infant) predict psychiatric disorders at age 7.5 years and to what extent does it predict different sub-groups?

## Methods

### Participants

Our sample was selected from participants in the Avon Longitudinal Study of Parents and Children (ALSPAC). ALSPAC is an ongoing population-based study which is studying a broad range of environmental, genetic and other influences on the health and development of children [28–30]. Pregnant women who were resident in the former Avon Health Authority in South-West England and had an estimated date of delivery between 1 April 1991 and 31 December 1992 were invited to take part. Of an initial 14,541 pregnancies enrolled, 13,988 singletons/twins were alive at 12 months of age. In the present study the sample was drawn from an approximate 10% sample of the core ALSPAC cohort who were invited to attend “Children in Focus” clinics after birth. When infants were 12 months old, 1,240 participating families (typically mother-infant dyads) attended the clinic.

At the clinic, numerous assessments were performed including the Thorpe Interaction Measure (TIM) [31]. The TIM involves the caregiver and child looking at a picture book. During this activity the caregivers are asked to interact with their child as they would if they were at home. All of the caregiver-infant interactions occurred in a ‘living room’ style environment in the clinic and were video recorded. The caregiver was told to stop when the infant started to lose interest in the activity or became distressed, and the video recording was then terminated.

Our sample included 180 mother-infant videos featuring children who went on to have a psychiatric assessment at age 7 years old using the Development and Wellbeing Assessment (DAWBA) [32]. This sample included all 60 children who went on to get a psychiatric diagnosis, along with 120 sex-matched controls randomly selected by the ALSPAC team. The diagnostic categories used in this assessment were based on the DSM-IV classification. Of the 180 videos, there were 176 videos that recorded vocalisations of sufficient quality for 176 children, 169 mothers and 15 fathers, with both parents attending the Child in Focus clinic in eight instances. Fifteen video recordings were excluded where the father was in attendance and a further three video recordings were excluded where the child did not vocalize, leaving 158 videos of individual mother and child pairs for analysis. The mother–infant interactions had a mean audio duration of 258 (standard deviation 153) seconds.

**Fig 1:**
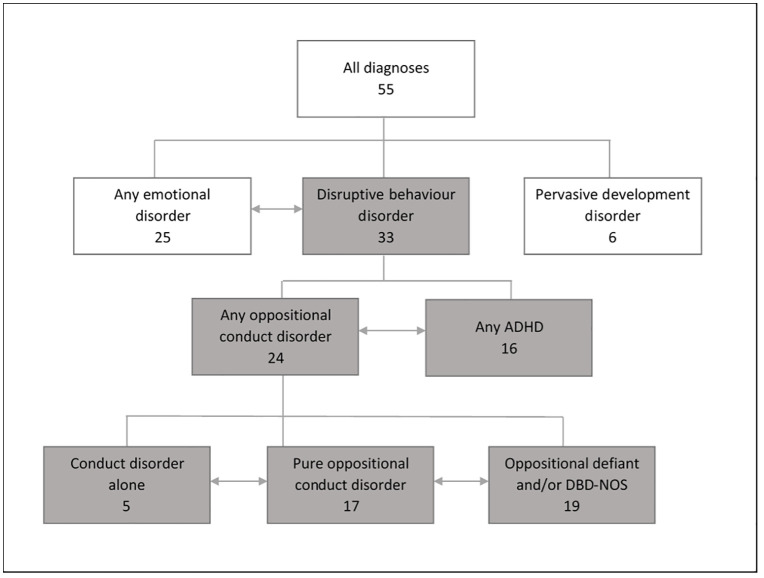
Flow diagram showing structure of the diagnostic categories of the cases for analysis. There is an element of overlap between groups of comorbidities presented (for example, nine infants are diagnosed with both any emotional disorder and DBD).

Fifty-five of the videos were of infants who were later given a psychiatric diagnostic categorisation of at least one of the following: pervasive development disorder (PDD) (autism), disruptive behaviour disorders (DBD, including attention-deficit/hyperactivity disorder (ADHD), conduct disorder, oppositional-defiant disorder, and/or DBD-not otherwise specified (NOS)), or any emotional disorder (anxiety or depressive disorder). The 55 infants in these videos are our cases for this study and Fig 1 outlines the hierarchy of the later psychiatric sub-diagnoses for these cases.

Ethical approval for the study was obtained from the ALSPAC Ethics and Law Committee and the Local Research Ethics Committees. Informed consent for the use of data collected via questionnaires and clinics was obtained from participants following the recommendations of the ALSPAC Ethics and Law Committee at the time. Please note that the study website contains details of all the data that is available through a fully searchable data dictionary and variable search tool [25].

### Procedure

Audio files were extracted from the video files and were used for the analysis. Assessors were blind to case or control status. The statistical software program R for Windows version 4.0.0 was used for all analyses and the package logistf was used for the Firth’s logistic regression [33]. Baseline characteristics were selected based on those used in previous publications using the same dataset [34] and examining vocalisation [27]. Potential predictors were: child gender, birth weight, and weight, length and ponderal index at 12 months; parental social status (defined by employment type), maternal age at birth, depression measured using the Edinburgh Postnatal Depression Scale (EPDS) [35] at 32–40 weeks gestation and at 8 months postnatally, and length of the pregnancy. These factors were selected as potential risk factors in infant diagnosis.

### Statistical methods

**Fig 2:**
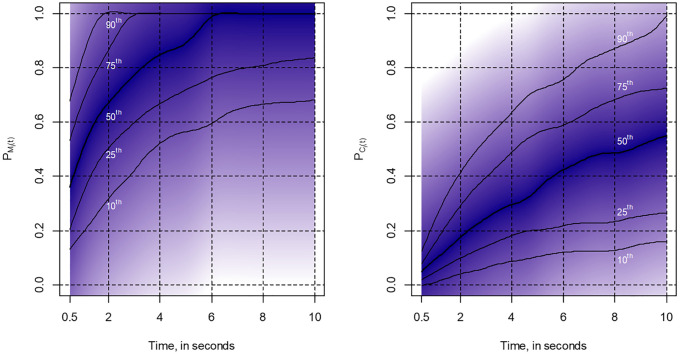
Estimated quantiles of the probability of mother response within t seconds, P_Mi_(t), and the probability of child response within t seconds, P_Ci_(t), for t between 0.5 and 10 seconds. Estimates smoothed using a cubic smoothing spline.

The length of the audio time of each video (seconds), the total duration of vocalisations (seconds) and the number of discrete vocalisations by both the child and mother had previously been recorded [27]. In this study, vocalisation start and stop times were used to construct time-to-response datasets for each of the mother-child pairs; one to estimate the time taken by the mother to respond to vocalisations by the child, and one to estimate the response time of the child to the mother’s vocalisations. Using survival analysis methods, specifically Kaplan-Meier curves, we were able to estimate the probability of the mother and child responding to each other within any specified time, T. We explored the predictive utility of a range of possible values for T by assessing the distribution of the probability of response for mother and child, and by looking at the estimated associations between overall caseness and the probability of response, between 0.5 and 10 seconds. We chose to use the probability of response within 1 second and within 8 seconds as a measure of vocal response for mothers and children respectively for subsequent analyses. These measures were deemed most useful since the distributions for the probability of responding for these values of T were relatively symmetrical and gave probability values that were not clustered near to the boundaries of 0 and 1. The estimated percentiles (10^th^, 25^th^, 50^th^, 75^th^ and 90^th^) of the probability of mother and child response are shown in Fig 2 and it can be seen that the 50^th^ percentile approximately aligns with 1 second for the mother and 8 seconds for the child and the distribution of the percentiles are symmetrical around these time points respectively. The association between overall caseness and probability of response was relatively stable for these values of T, which also confirmed our choice further (see S1 File, and the embedded S1-S5 Figs Supporting Information, for further detail on choice of these metrics).

Univariate linear regression models were used to assess the association between mother and child baseline characteristics and the outcomes, probability of response of the mother and the child within 1 and 8 seconds respectively. For each baseline characteristic modelled, the beta coefficient estimate and 90% confidence interval are presented for each of the outcomes. The beta coefficients for continuous measures reflect the change in the outcome measure for every 1-unit increase in the baseline characteristic modelled.

We used logistic regression to estimate the association between the probability of response of the mother and child and caseness, overall and by diagnostic subgroup. Due to the low numbers for some diagnoses, some models produced unstable estimates and so Firth’s penalized-likelihood logistic regression was used to reduce bias in the estimates for all diagnostic outcome models. Initial models looked at mother and child probability of response separately with adjustments for baseline characteristics associated with the respective probability of response measure. As a sensitivity analyses, we further adjusted to include both the mother and child probability of response measures in addition to baseline characteristics associated with vocalisation responsiveness of the child and mother. As an additional analysis, we similarly estimated the associations between the probability of response and caseness overall by child sex. Model fit was assessed using the Hosmer-Lemeshow test and Likelihood Ratio tests. Odds ratio estimates, 95% confidence intervals and p-values are presented.

We present the distribution of the probability of mother and child response measures within diagnostic subgroups using boxplots.

## Results

Table 1 presents the number of cases and controls for analysis and the number of children within each diagnostic subgroup summarised overall.

**Table 1:** Numbers of cases and controls, and the number in each diagnostic subgroup.

Variable	Total N
Number of Videos	158
Control	103
Case (Any Disorder)	55
Disruptive Behaviour Disorder (DBD)	33
Any Attention Deficit Hyperactivity Disorder (ADHD)	16
Pervasive Development Disorder (Autism)	6
Any Emotional Disorder	25

Table 2 shows the findings of a series of univariate linear regression models performed to assess associations between baseline characteristics and probability of vocal response for mothers and children. No baseline characteristics were found to be associated with child vocalisation responsiveness. Children’s birth weight and mother’s age at the birth of the child were found to be associated with the mother’s vocal response measure. An increase of 7.2% (90% Confidence Interval (CI): 0.9–13.5%) was seen in the probability of mother response within 1 second for every one kilogram increase in the child’s birthweight and an increase of 1.1% (90% CI: 0.4–1.9%) was seen for every 1 year increase in the mother’s age at delivery.

**Table 2:** Associations between mother and child baseline characteristics, and probability of response metrics, probability of response of the mother within 1 second and probability of response of the child within 8 seconds.

Predictor variable	Probability of response within t seconds	
	Child (t = 8)	Mother (t = 1)
Child gender (Male vs. Female)	0.037 (−0.040, 0.115)	−0.054 (−0.125, 0.017)
Mother social class (Classes IIIM, IV or V vs. I, II or IIINM)	0.108 (−0.002, 0.218)	−0.079 (−0.177, 0.018)
Child birthweight (kg)	0.036 (−0.033, 0.105)	0.072 (0.009, 0.135) *
Child weight at 12 months (kg)	0.012 (−0.017, 0.041)	0.016 (−0.011, 0.042)
Child length at 12 months (cm)	0.006 (−0.007, 0.020)	0.004 (−0.009, 0.017)
Child ponderal index at 12 months (g/cm³)	0.000 (−0.020, 0.019)	0.007 (−0.011, 0.025)
Mother age at birth (years)	0.006 (−0.002, 0.014)	0.011 (0.004, 0.019) *
Maternal depression at 32–40 weeks gestation	0.006 (−0.002, 0.013)	0.005 (−0.002, 0.012)
Maternal depression at 8 months postnatal	0.002 (−0.005, 0.009)	−0.001 (−0.008, 0.005)
Length of pregnancy (weeks)	0.003 (−0.019, 0.025)	0.003 (−0.017, 0.023)

Beta coefficient estimates from univariate linear regression models, with corresponding 90% confidence intervals. Estimates are shown per 1-unit increase for predictors measured on a continuous scale. * indicates confidence intervals that exclude zero.

As shown in Table 3, neither overall caseness, nor any diagnostic subgroup, was associated with child vocal response with the exception of ADHD, where we estimated a 21% (95% CI: 1–48%) increase in the odds of receiving this diagnosis for every 10% increase in the probability of child response within 8 seconds. Mother’s vocalisation responsiveness, on the other hand, was found to be predictive of caseness overall, and of DBD, any ADHD, but not PDD or any emotional disorder. For every 10% increase in the probability of mother response within 1 second, we estimated a 17% (95% CI: 5–29%) decrease in the odds of the child receiving any disorder diagnosis. It made little difference whether vocal response measures were examined individually or within the same model.

**Table 3:** Associations between probability of response of the mother and child, and overall caseness (any disorder) and specific diagnoses.

Disorder	Model	Probability of Child Response within 8 Seconds	Probability of Mother Response within 1 Second
		Estimate, 95% CI and P-value	Estimate, 95% CI and P-value
Any Disorder	Initial	1.11 (0.98, 1.25), 0.090	0.83 (0.71, 0.95), 0.010 *
	Extended	1.09 (0.96, 1.23), 0.177	0.84 (0.72, 0.96), 0.015 *
Any Attention Deficit Hyperactivity Disorder (ADHD)	Initial	1.21 (1.01, 1.48), 0.046 *	0.79 (0.63, 0.99), 0.039 *
	Extended	1.21 (0.99, 1.49), 0.053	0.79 (0.61, 1.00), 0.048 *
Disruptive Behaviour Disorder (DBD)	Initial	1.15 (1.00, 1.33), 0.054	0.80 (0.67, 0.94), 0.009 *
	Extended	1.14 (0.98, 1.32), 0.081	0.80 (0.67, 0.95), 0.013 *
Pervasive Development Disorder (Autism)	Initial	1.09 (0.82, 1.46), 0.533	1.02 (0.73, 1.42), 0.913
	Extended	1.07 (0.80, 1.42), 0.611	1.03 (0.74, 1.46), 0.851
Any Emotional Disorder	Initial	1.00 (0.86, 1.17), 0.965	0.89 (0.74, 1.06), 0.187
	Extended	0.99 (0.84, 1.15), 0.860	0.89 (0.74, 1.06), 0.183

Odds ratios associated with a 10% increase in the probability of responding within 8 seconds for children and within 1 second for mothers, 95% confidence intervals and p-values are displayed. Initial models include either the probability of child or mother response as a predictor and are adjusted for any baseline characteristics associated with the probability of response measure in the model, specifically child’s birthweight and mother’s age at birth for the probability of mother response but no adjustments for the probability of child response. Extended models include both the probability of child and mother response as predictors and are adjusted for baseline characteristics associated with both the probability of child and mother response. * indicates confidence intervals that exclude zero.

We looked at the associations between vocal response measures separately for male and female children (Table 4). For the probability of the mother responding within 1 second, there was no evidence that the association differed by child sex (p for interaction 0.52). However, for the probability of the child responding within 8 seconds, there was some evidence of a difference between boys and girls (p for interaction 0.085), with no evidence of an association for girls, but a positive association for boys, such that every 10% increase in the probability of a male child responding within 8 seconds, we estimated around an 18% (95% CI: 1–38%) increase in the odds of the child receiving any disorder diagnosis by the age of 7. This is a weak finding in a subgroup analysis, but may warrant further investigation.

**Table 4:** Associations between the probability of response of the mother and child, and overall caseness (any disorder) for males and females separately.

Disorder	Model	Probability of Child Response within 8 Seconds	Probability of Mother Response within 1 Second
		Estimate, 95% CI and P-value	Estimate, 95% CI and P-value
Any Disorder (Males)	Initial	1.19 (1.03, 1.40), 0.021 *	0.82 (0.68, 0.97), 0.025 *
	Extended	1.18 (1.01, 1.38), 0.038 *	0.82 (0.67, 0.98), 0.034 *
Any Disorder (Females)	Initial	0.95 (0.76, 1.17), 0.640	0.87 (0.67, 1.09), 0.231
	Extended	0.93 (0.73, 1.17), 0.549	0.86 (0.67, 1.08), 0.208

Odds ratios associated with a 10% increase in the probability of responding within 8 seconds for children and within 1 second for mothers, 95% confidence intervals and p-values are displayed. Initial models include either the probability of child or mother response as a predictor and are adjusted for any baseline characteristics associated with the probability of response measure in the model, specifically child’s birthweight and mother’s age at birth for the probability of mother response but no adjustments for the probability of child response. Extended models include both the probability of child and mother response as predictors and are adjusted for baseline characteristics associated with both the probability of child and mother response. * indicates confidence intervals that exclude zero.

**Fig 3:**
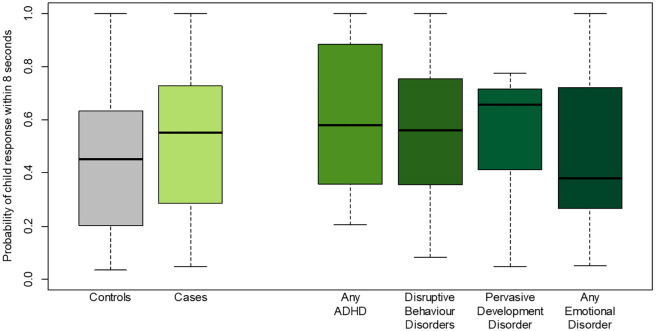
Distribution of the probability of child response within 8 seconds amongst controls, cases and each diagnostic subgroup. Each box represents the median and upper/lower quartiles with the whiskers showing the 5th and 95th percentiles, and any points beyond this range shown individually.

**Fig 4:**
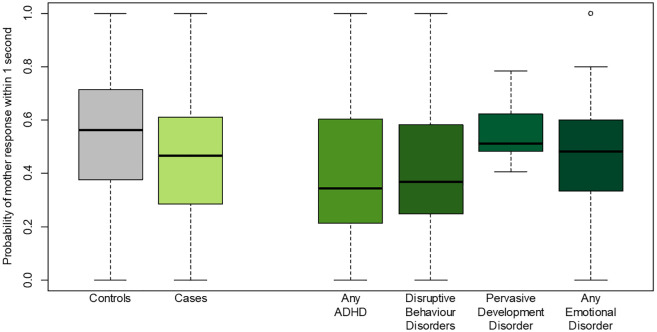
Distribution of the probability of mother response within 1 second amongst controls, cases and each diagnostic subgroup. Each box represents the median and upper/lower quartiles with the whiskers showing the 5th and 95th percentiles, and any points beyond this range shown individually.

Fig 3 and Fig 4 present the distribution of vocal response across cases, controls and the individual diagnostic subgroups for children and mothers respectively. Lower levels of maternal vocal response (i.e., lower probability of response within 1 second) can be seen in all cases compared to controls, particularly for diagnoses of DBD and any ADHD.

## Discussion

The current study examined the probability of vocal response during parent-infant interactions and later psychiatric diagnosis. In this sample of mother-infant dyads, the data suggest that a lower probability of maternal vocal response may be associated with an increased likelihood that infants are later diagnosed, at age seven years, with at least one psychiatric disorder, including DBD or any ADHD. However, these findings should be interpreted with considerable caution. The diagnostic subgroups were small (e.g., only six cases of pervasive developmental disorder), limiting statistical power and increasing the risk that observed associations may be unstable or non-generalisable.

The exploratory findings reported here complement our earlier results based on this dataset suggesting that lower levels of maternal engagement with an infant are associated with later DBDs including ADHD and conduct disorder, but not with autism or emotional disorders. These findings were consistent across a range of variables including maternal physical activity, positive parenting behaviours and frequency and duration of vocalisation [4–6,27,36,37]. As in the earlier ALSPAC studies, infant behaviours were not found to predict later diagnoses.

The lack of association between child probability of vocal response and later autism is inconsistent with findings from previous studies. For instance, infants and toddlers with autism have been shown to produce fewer speech-related vocalisations and fewer vocal interactions with adults when compared to typically developing children [18,38]. However, another finding in the present study was consistent with earlier studies. Specifically, we found that the infants who were later diagnosed with autism engaged in more “monologues”. In other words, they engaged in more vocalisations which were not accompanied by or responded to by the caregiver. The lack of response to the child by the caregiver might indicate that “they were often non-social or at least unrelated to what the adult in the setting was doing” [18]. Previous studies have found that the mothers of children with ASD spoke less [39,40].

Parental factors such as maternal unresponsiveness may be one of the potential mechanisms underlying the association between increased infant vocalisation and the later development of disruptive behaviour disorders [10,41]. Other potential explanations may be differences in the attachment relationship between mother and child [42], genetic factors or gene–environment interactions [43,44]. Socioeconomic status did not explain the association which was found between altered maternal vocalisation frequencies and later diagnosis of child psychopathology [27]. Previous studies have found socioeconomic status is associated with quantity and quality of parental speech directed to their children [45].

### Limitations

One of the main limitations of the present study is the relatively small diagnostic sub-groups. For instance, there were only six cases of pervasive developmental disorder. As such, the results should be viewed as preliminary and exploratory, and not as evidence for any definitive causal or predictive relationship. Regarding the vocalisations, because the video quality was so poor, we cannot be certain that these were actual responses to the previous vocalisation. Additionally, the analysis in the present study focused only on vocal response (i.e., the time between the end of one person vocalising and the other starting) and did not assess the quality or sequential patterns of vocalisation between the caregiver and infant [46]. An extended analysis of intonation and content could have been informative but the sound quality on the videos, while adequate for the analysis performed in the present study, was not sufficient to allow for more detailed linguistic and pitch contour analysis. It is possible that there are cases within the control group and vice versa [27]: for example ADHD may commonly be under- and over-diagnosed [47]. Moreover, there are substantial limitations with the quality of the video material and extracted sound files. The videos used in the present study, recorded in a laboratory setting, may not accurately represent typical vocal behaviour in both the caregiver and the infant.

### Clinical implications

Attempting to identify early predictors (or potential clinical markers) in the vocalisations between caregivers and infants of later childhood psychiatric disorder is important as such efforts have the potential in the future for optimising the development of systems for the early detection of developmental anomalies [48]. It is possible that the behaviour problems most easily identifiable in this dataset, namely ADHD and conduct disorders, may be more amenable to early intervention than other psychiatric disorders [48,49].

### Future research directions

Future studies could investigate the relative effects of genetic and behavioural contributions made by parental diagnosis (such as autism or ADHD) on the child’s psychopathology. Additionally, caregivers tend to use infant-directed speech which is characterised by utterances which are slower, simplified, “stretched” temporally and spectrally, and exaggerated prosocially [49]. A substantial body of research has shown that infants have a tendency to respond much more strongly to parents’ infant-directed speech when compared with adult-directed speech [50]. Future studies should investigate not only vocal response and frequency but also explore whether exaggerated pitch contours (and other features of infant-directed speech) play a contributory role in being able to predict later childhood psychiatric disorders.

## Conclusion

These findings demonstrate the potential clinical value of the analysis of caregiver–infant vocalisations in predicting later development of childhood psychiatric disorders. It is hoped that analyses of this kind can contribute to the development of screening instruments for disorders which are amenable to early intervention [51].

## Supporting information

S1 File Justification for choice of vocal response metrics. (DOCX)

S1 Fig Distribution of the probability of mother response within t seconds, P_Mi_(t), and the probability of child response within t seconds, P_Ci_(t), for t = 1, 5 and 10 seconds. (TIF)

S2 Fig Estimated quantiles of the probability of mother response within t seconds, P_Mi_(t), and the probability of child response within t seconds, P_Ci_(t), for t between 0.5 and 10 seconds. Estimates smoothed using a cubic smoothing spline.
(TIF)

S3 Fig Estimated mean and standard deviation of the probability of mother response within t seconds, P_Mi_(t), and the probability of child response within t seconds, P_Ci_(t), for t between 0.5 and 10 seconds. Estimates smoothed using a cubic smoothing spline.
(TIF)

S4 Fig Estimated skewness of the probability of mother response within t seconds, P_Mi_(t), and the probability of child response within t seconds, P_Ci_(t), for t between 0.5 and 10 seconds. Estimates smoothed using a cubic smoothing spline.
(TIF)

S5 Fig Estimated associations between the probability of mother response within t seconds, P_Mi_(t), and the probability of child response within t seconds, P_Ci_(t), and overall caseness, for t between 0.5 and 10 seconds. Models fitted using Firth’s penalised logistic regression, adjusted for child gender. Association shown as odds ratio, with 95% confidence limits. Estimates smoothed using a cubic smoothing spline.
(TIF)
