# Supplementary material for: Probability of a timely vocal response in mother-infant interaction and later psychiatric diagnosis: A case-control study
Source: PLoS One. 2026 Jul 1;21(7):e0344552. doi: 10.1371/journal.pone.0344552 (PMC13322543; doi:10.1371/journal.pone.0344552)
Supplement: S1 File — (DOCX) [file pone.0344552.s001.docx]

**Justification for choice of metric of child and maternal vocal responsiveness**

For each mother-child conversation, we are interested in the time it takes for the mother to respond to the child, and for the child to respond to the mother.

If we define S_Mi_(t) as the survivor function for the i^th^ mother, then S_Mi_(t) is the probability that the i^th^ mother takes at least t seconds to reply to the child. P_Mi_(t) = 1 - S_Mi_(t) is the probability that the i^th^ mother responds within t seconds.

Similarly, S_Ci_(t) is the probability that the i^th^ child takes longer than t seconds to respond, and P_Ci_(t) = 1 - S_Ci_(t) is the probability that the i^th^ child responds within t seconds.

S1 Supplementary Figure shows the distribution of P_Mi_(t) and P_Ci_(t) for t=1, t=5, and t=10 seconds.

| S1 Supplementary Figure: Distribution of P_Mi_(t) and P_Ci_(t), for t=1, 5, and 10 seconds. | | |
| --- | --- | --- |
| Histogram of P_Mi_(1) | Histogram of P_Mi_(5) | Histogram of P_Mi_(10) |
| Histogram of P_Ci_(1) | Histogram of P_Ci_(5) | Histogram of P_Ci_(10) |

S2 Supplementary Figure shows the distribution of P_Mi_(t) and P_Ci_(t), in terms of the 10^th^, 25^th^, 50^th^, 75^th^, and 90^th^ percentiles, for all values of t between 0.5 and 10 seconds.

| S2 Supplementary Figure. Estimated quantiles of P_Mi_(t) and P_Ci_(t), for t between 0.5 and 10 seconds. Estimates smoothed using a cubic smoothing spline. | |
| --- | --- |
| Graph showing P_Mi_(t)  vs. t | Graph showing P_Ci_(t)  vs. t |

S3 Supplementary Figure and S4 Supplementary Figure show additional measures of the distribution of P_Mi_(t) and P_Ci_(t), namely, the mean and standard deviation, and skewness respectively.

| S3 Supplementary Figure. Estimated mean and standard deviation (SD) of P_Mi_(t) and P_Ci_(t), for t between 0.5 and 10 seconds. Estimates smoothed using a cubic smoothing spline. | |
| --- | --- |
| Graph showing mean and SD of P_Mi_(t)  vs. t | Graph showing mean and SD of P_Ci_(t)  vs. t |

| S4 Supplementary Figure. Estimated skewness of P_Mi_(t) and P_Ci_(t), for t between 0.5 and 10 seconds. Estimates smoothed using a cubic smoothing spline. | |
| --- | --- |
| Graph showing skewness of P_Mi_(t)  vs. t | Graph showing skewness of P_Ci_(t)  vs. t |

These figures suggest that for P_Mi_(t), values of t between 1 and 2 will be most useful as a measure of vocal responsiveness for mothers, as these times give values that are not clustered near to the boundaries of 0 and 1, and yield a relatively symmetrical distribution. Similarly, values of t between 6 and 10 will generate a distribution of P_Ci_(t) that is most suitable as a measure of vocal responsiveness for children.

S5 Supplementary Figure shows the estimated association between P_Mi_(t) and overall caseness, and the association between P_Ci_(t) and overall caseness, for values of t between 0.5 and 10 seconds; all models are fitted using Firth’s penalised logistic regression, adjusted for child gender.

| S5 Supplementary Figure. Estimated associations between P_Mi_(t) and P_Ci_(t), with overall caseness, for t between 0.5 and 10 seconds. Models fitted using Firth’s penalised logistic regression, adjusted for child gender. Association shown as odds ratio (OR), with 95% confidence interval (CI). Estimates smoothed using a cubic smoothing spline. | |
| --- | --- |
| Graph showing OR and 95% CI for P_Mi_(t) vs. t | Graph showing OR and 95% CI for P_Ci_(t) vs. t |

These figures show that the estimated association between P_Mi_(t) with overall caseness is relatively stable for values of t below 6 seconds. The estimated association between P_Ci_(t) with overall caseness is relatively stable for values of t above 6 seconds.

Taking all of these factors into consideration, we chose to measure mother and child vocal responsiveness using P_Mi_(1) and P_Ci_(8).
