# Supplementary figures and images for: Probability of a timely vocal response in mother-infant interaction and later psychiatric diagnosis: A case-control study
Source: PLoS One. 2026 Jul 1;21(7):e0344552. doi: 10.1371/journal.pone.0344552 (PMC13322543; doi:10.1371/journal.pone.0344552)

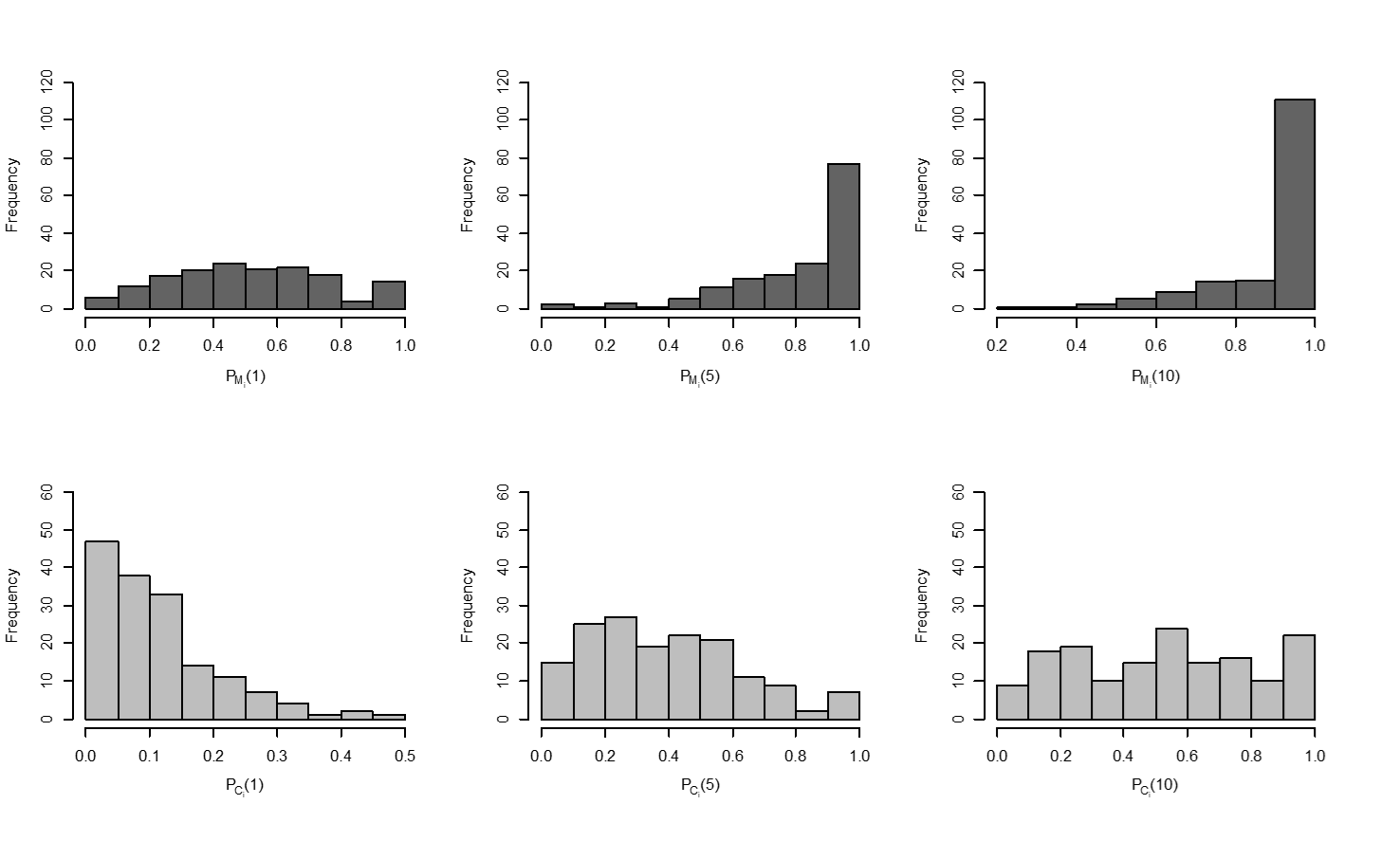

Supplement: S1 Fig — (TIF) [file pone.0344552.s002.tif]

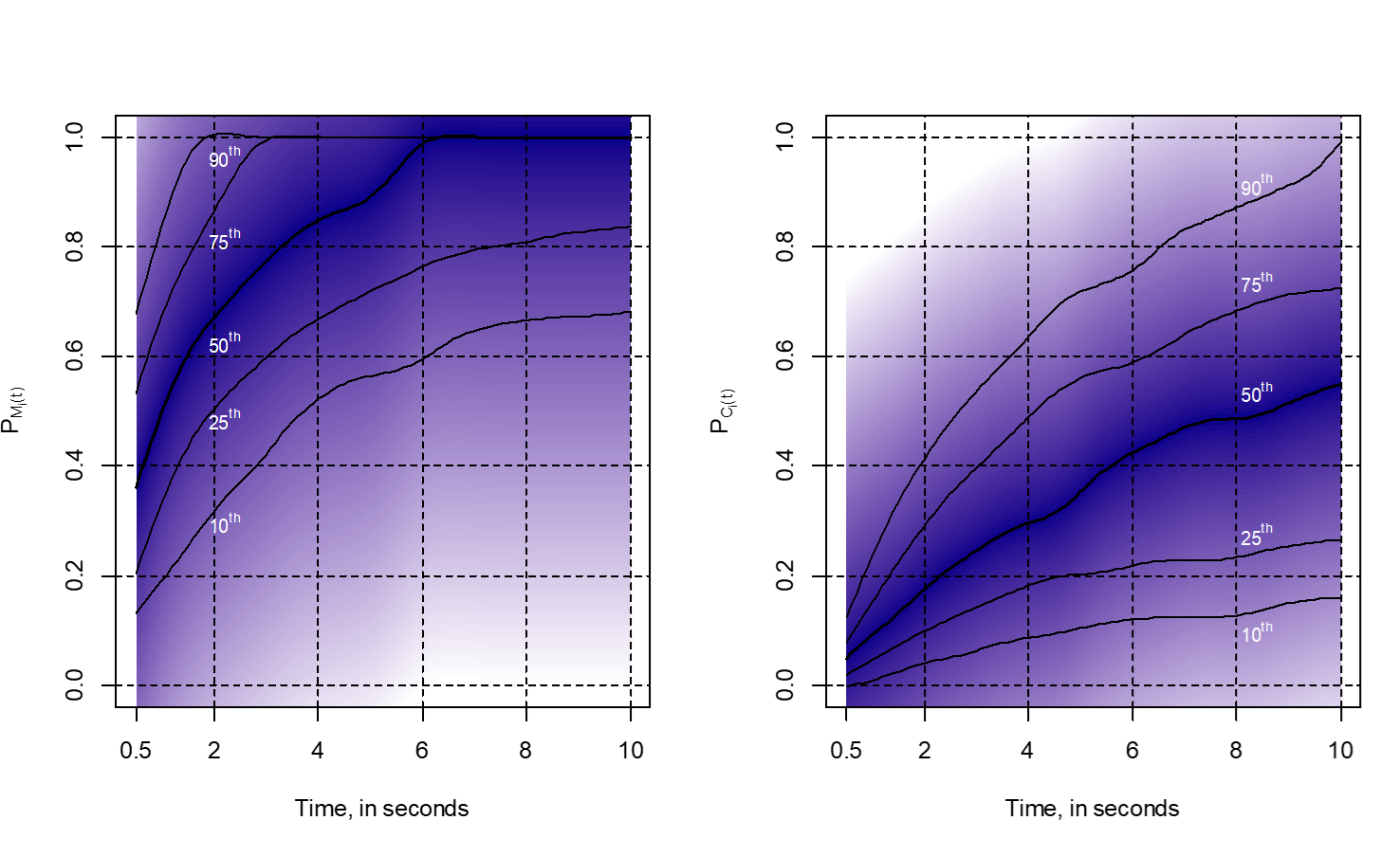

Supplement: S2 Fig — Estimates smoothed using a cubic smoothing spline. (TIF) [file pone.0344552.s003.tif]

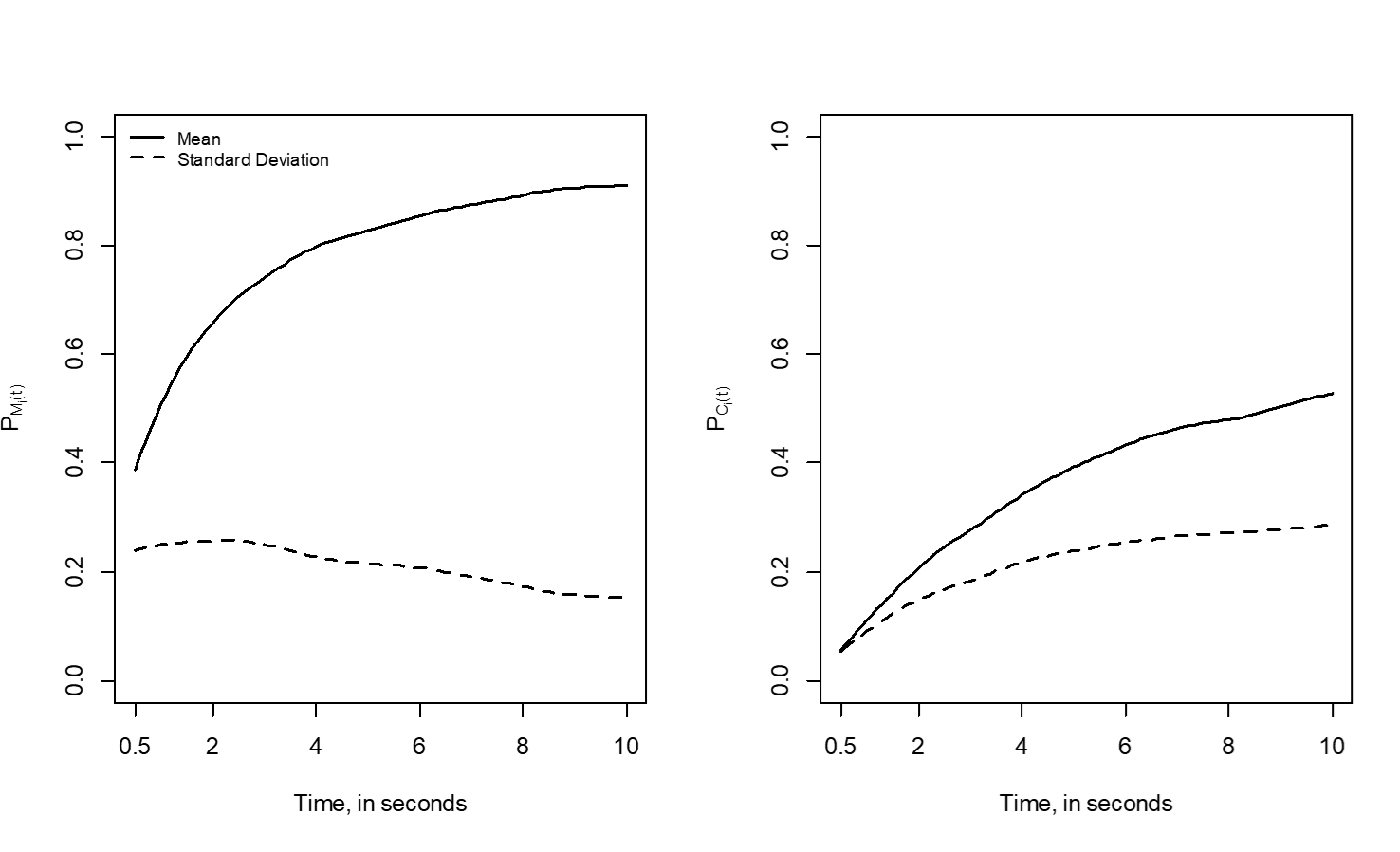

Supplement: S3 Fig — Estimates smoothed using a cubic smoothing spline. (TIF) [file pone.0344552.s004.tif]

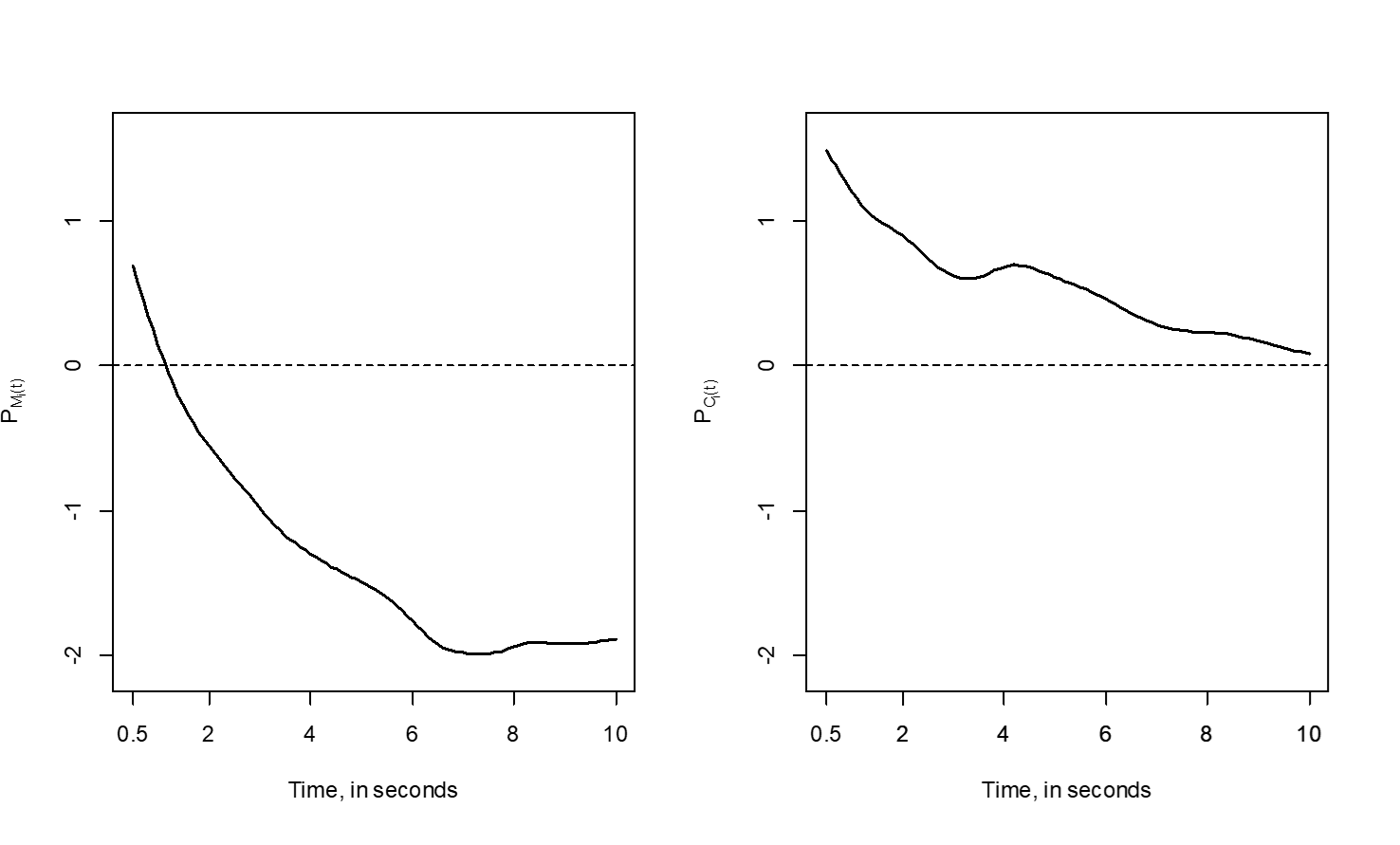

Supplement: S4 Fig — Estimates smoothed using a cubic smoothing spline. (TIF) [file pone.0344552.s005.tif]

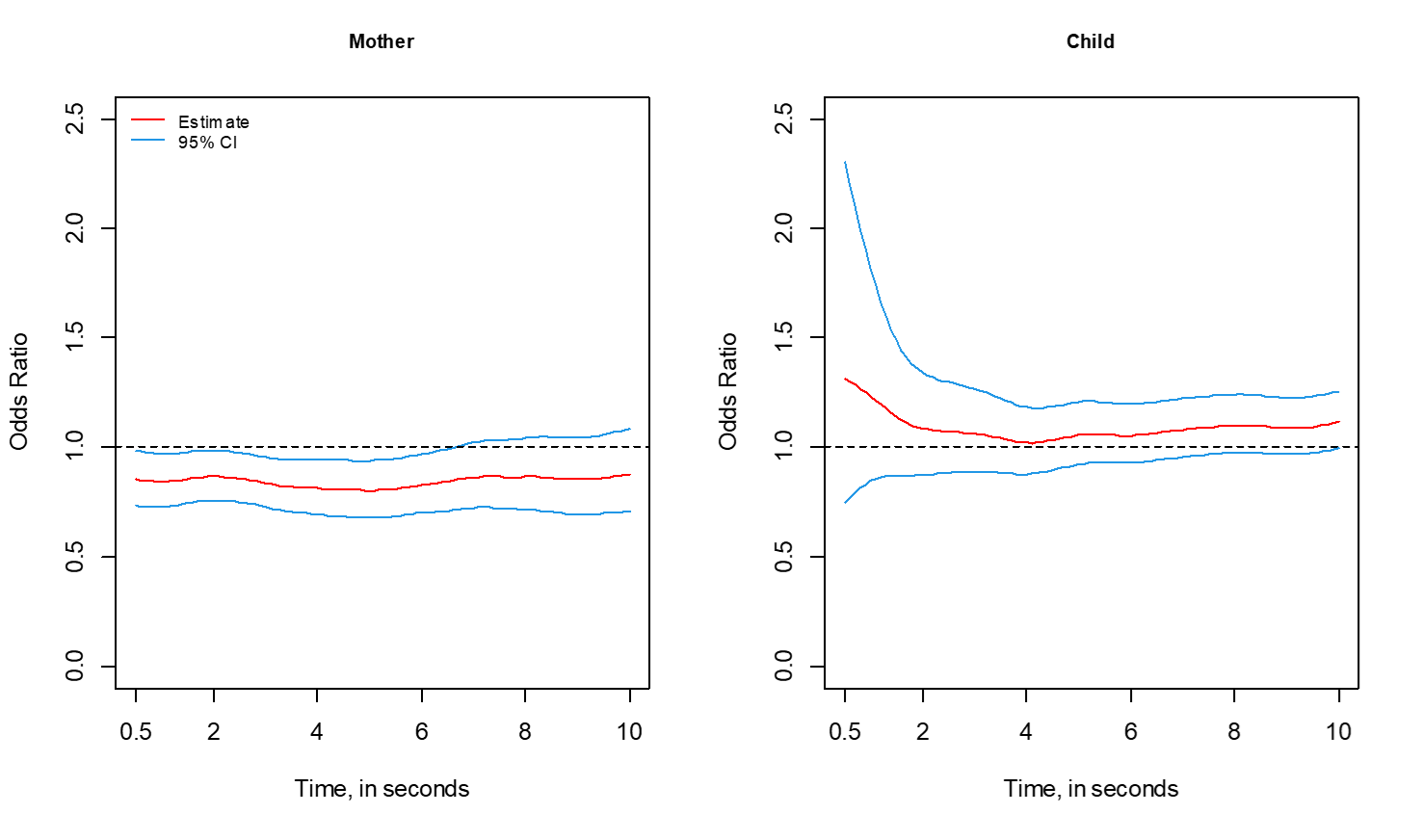

Supplement: S5 Fig — Models fitted using Firth’s penalised logistic regression, adjusted for child gender. Association shown as odds ratio, with 95% confidence limits. Estimates smoothed using a cubic smoothing spline. (TIF) [file pone.0344552.s006.tif]
